# Closed‐loop insulin delivery in end‐of‐life care: a case report

**DOI:** 10.1111/dme.13974

**Published:** 2019-05-03

**Authors:** C. K. Boughton, L. Bally, S. Hartnell, M. Wilinska, A. P. Coll, M. Evans, C. Stettler, R. Hovorka

**Affiliations:** ^1^ Wellcome Trust‐Medical Research Council Institute of Metabolic Science University of Cambridge Cambridge UK; ^2^ Bern University Hospital Department of Diabetes Endocrinology Clinical Nutrition and Metabolism Bern Switzerland; ^3^ Cambridge University Hospitals NHS Foundation Trust Wolfson Diabetes and Endocrine Clinic Cambridge UK

## Abstract

**Background:**

Glucose management for people with diabetes approaching the end of life can be very challenging. The aim is to balance a minimally invasive approach with avoidance of symptomatic hypo‐ and hyperglycaemia.

**Case report:**

We present a case of a hospitalized individual whose glucose was managed with closed‐loop insulin delivery within a randomized controlled trial setting during a period of terminal illness. During the time in which closed‐loop insulin delivery was used, glucose control was safe, with no glucose‐related harm. The mean ± sd sensor glucose for this individual was 11.3 ± 4.3 mmol/l, percentage of time spent in target glucose range between 6 and 15 mmol/l was 70.5%, time spent in hypoglycaemia was 2.0% and time spent in significant hyperglycaemia >20 mmol/l was 2.6%.

**Conclusion:**

Closed‐loop systems can accommodate personalized glucose targets and highly variable insulin requirements. Factory‐calibrated continuous glucose sensors and insulin pump therapy are less intrusive than finger‐stick glucose measurements and insulin injections, respectively. Closed‐loop systems may provide a safer and less burdensome approach to glucose management towards the end of life.

## What's new?

- Glucose management for people with diabetes approaching the end of life can be very challenging, and clinical practice is variable.
- We present a case where closed‐loop insulin delivery was used to manage glucose levels in an individual hospitalized during a period of terminal illness.
- During the time in which closed‐loop insulin delivery was used, glucose control was safe, with no glucose‐related harm.
- Closed‐loop systems may provide a safer and less burdensome approach to glucose management towards the end of life.
- Studies are needed to determine safety, efficacy and acceptability in this population.

**What's new?**

- Glucose management for people with diabetes approaching the end of life can be very challenging, and clinical practice is variable.
- We present a case where closed‐loop insulin delivery was used to manage glucose levels in an individual hospitalized during a period of terminal illness.
- During the time in which closed‐loop insulin delivery was used, glucose control was safe, with no glucose‐related harm.
- Closed‐loop systems may provide a safer and less burdensome approach to glucose management towards the end of life.
- Studies are needed to determine safety, efficacy and acceptability in this population.

## Introduction

Diabetes is one of the commonest comorbidities in end‐of‐life care and management of blood glucose during this period can be challenging 1, 2. Pathophysiological changes occur towards the end of life, predisposing to accentuated perturbations of glucose levels; hyperglycaemia may result from the stress response to severe illness, use of steroids for symptom relief or co‐existent infections 3. Insulin requirements may fall as a result of reduced oral intake and weight loss, and renal or hepatic failure, with the consequent risk of hypoglycaemia 4, 5.

End‐of‐life diabetes care requires a holistic approach; blood glucose monitoring should be minimized and complex insulin regimens avoided. It is important, however, to maintain adequate control to enhance comfort by preventing hyperglycaemia‐induced thirst, dehydration, confusion, drowsiness and symptomatic hypoglycaemia 2. Clinical recommendations are not evidence‐based but recommend target glucose levels between 6 and 15 mmol/l, with as minimally invasive an insulin regimen and as infrequent monitoring as possible to keep the person comfortable without compromising safety 4, 6. Despite these recommendations, clinical practice is highly variable 2, 4.

The role of diabetes technologies in improving the quality and experience of diabetes care for patients and relatives towards the end of life has not been determined. Closed‐loop systems, which combine real‐time glucose measurements from a continuous glucose monitor with a control algorithm that directs insulin delivery via an insulin pump have been shown to be safe and effective in the inpatient setting 7, 8. Closed‐loop systems, which automatically deliver insulin in a glucose‐responsive manner, may provide a solution to glucose management for people hospitalized towards the end of life.

In this case report we describe the case of a participant in a randomized controlled trial whose glucose was managed safely with a closed‐loop system during a period of terminal illness.

## Case report

The individual in this case was a 79‐year‐old woman who presented with vomiting, increased stoma output and vertigo. She had a medical history of surgically resected bowel cancer, hypothyroidism, deep vein thrombosis treated with tinzaparin, and Type 2 diabetes mellitus managed with oral hypoglycaemic agents (metformin and linagliptin). Prior to admission she was independent in activities of daily living. A computerized tomography (CT) scan at presentation showed a large posterior fossa haemorrhage, which was managed with reversal of tinzaparin and blood pressure control. Metformin was stopped on admission as a result of dehydration from increased stoma output, and the woman received corrective doses of aspart as required to treat hyperglycaemia.

Subsequently, the woman developed increased confusion and drowsiness, and repeat CT scan showed mass effect with hydrocephalus. She was transferred to the regional neurosurgical unit and an external ventricular drain inserted. Hyperglycaemia was initially managed with a variable rate intravenous insulin infusion with 2–4 units/h, but hyperglycaemia persisted (capillary blood glucose levels 12–16 mmol/l). Nasogastric feeding was commenced because of fluctuating consciousness [Glasgow Coma Scale 8–14] with Nutrison protein Plus 50 ml/h for 20 h (7.1 g carbohydrate/h), with a 4‐h break, and this was subsequently uptitrated to 75 ml/h (10.7 g carbohydrate/h).

### Randomized controlled trial setting

During the woman's stay in the hospital, we were undertaking a multinational randomized controlled trial contrasting fully closed‐loop insulin delivery with faster‐acting insulin aspart (Fiasp; Novo Nordisk, Bagsværd, Denmark), or conventional subcutaneous insulin therapy over a period of up to 15 days of hospital stay 9. In the UK, the study protocol was approved by the local Research Ethics Committees (East of England Central Cambridge Ethics Committee) and the Medicines and Healthcare products Regulatory Agency (MHRA).

Recruitment of this particular woman into the study was discussed with her family, and a consultee declaration form was signed by her next‐of‐kin. Although prognosis was guarded, there were no limits to treatment escalation at the time of recruitment into the study. The woman was randomized to closed‐loop insulin delivery. Pre‐study total daily insulin dose was 110 units.

### Closed‐loop insulin delivery

**Figure 1:**
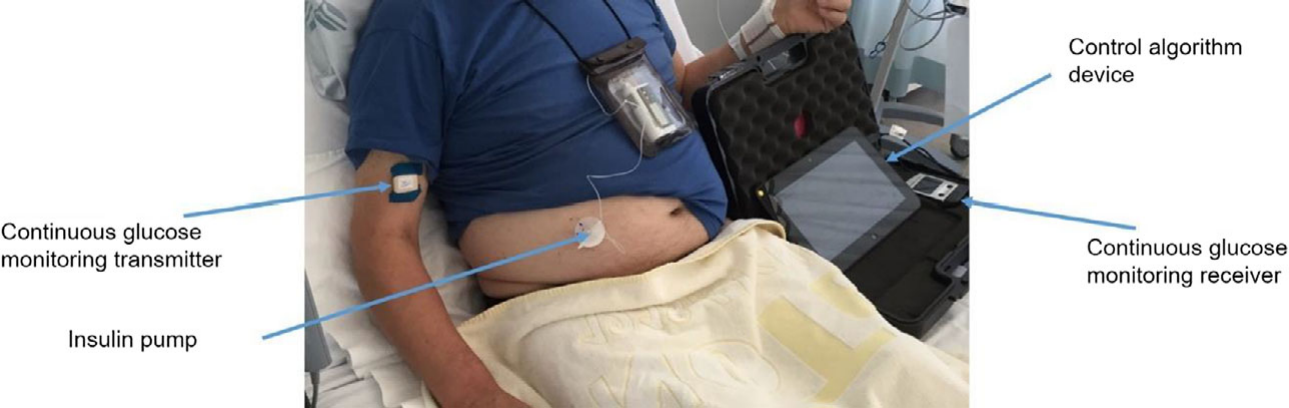
Automated fully closed‐loop insulin delivery prototype (FlorenceD2W‐T2) used by the woman in our case report (photo obtained with consent).

A subcutaneous cannula was inserted into the abdomen/arm for delivery of faster‐acting insulin aspart by insulin pump (Dana R Diabecare; SOOIL Development, Seoul, Korea). A subcutaneous real‐time continuous glucose monitor (Freestyle Navigator II; Abbott Diabetes Care, Alameda, CA, USA) was inserted into the abdomen/arm and calibrated according to manufacturer's instructions (Fig. 1).

The FlorenceD2W‐T2 closed‐loop system consisted of a model predictive control algorithm (version 0.3.70) residing on a control algorithm device (Dell Latitude 10 Tablet, Dell, UK) linked by a USB cable to the continuous glucose monitoring receiver (FreeStyle Navigator II). The tablet device communicated with the study pump via a Bluetooth wireless communication protocol. The control algorithm was initialized using the woman's weight and pre‐study total daily insulin dose. The control algorithm did not receive announcements regarding timing or carbohydrate content of meals, enteral or parenteral feeds. No adjustments to target glucose were made during the study.

The control algorithm adapted itself to a particular participant by updating model variables and refining participant's insulin requirements. The algorithm aimed to achieve glucose levels between 5.8 and 7.2 mmol/l, and adjusted the actual target level depending on accuracy of the model‐based glucose predictions and prevailing glucose levels. Safety rules limited maximum insulin infusion and suspended insulin delivery at sensor glucose ≤4.2 mmol/l, or when sensor glucose was rapidly decreasing. If sensor glucose was unavailable for 30 min, the research team was alerted and the study pump insulin infusion rate reverted to the pre‐programmed basal rate.

### Results

**Figure 2:**
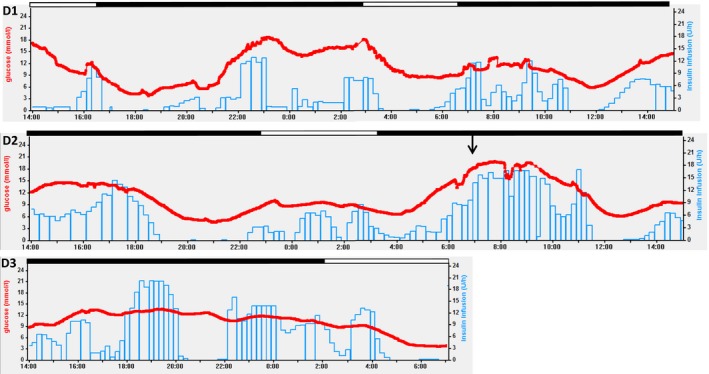
Sensor glucose levels (red line), closed‐loop algorithm directed insulin delivery (blue line) during the period of closed‐loop glucose control (D1, 2 and 3 are consecutive days of closed‐loop insulin delivery during the study). Black filled line above graph indicates ongoing nasogastric feeding and unfilled indicates feed discontinued. Arrow indicates clinical deterioration with hospital‐acquired pneumonia.

During the study period, after 2 days of closed‐loop insulin delivery, the woman developed hospital acquired pneumonia. Antibiotics, oxygen therapy and chest physiotherapy were commenced and closed‐loop was continued, with notably higher insulin requirements (Fig. 2). Despite optimal ward‐based treatment, her clinical condition deteriorated and clinical focus switched to palliation; the nasogastric feed was stopped and, as insulin requirements were minimal without the feed, closed‐loop and participation in the study were stopped at this point. A syringe driver was started for symptom control and she died several days later. The death was reported to the trial Data and Safety Monitoring Board as an adverse event unrelated to study devices and procedures.

During the period of closed‐loop insulin delivery, glucose control was safe with no glucose‐related harm. The mean sensor glucose was 11.3 ± 4.3 mmol/l and percentage of time with sensor glucose between 6 and 15 mmol/l was 70.5%. Time spent in hypoglycaemia <3.9 mmol/l was 2.0% and time spent in significant hyperglycaemia >20 mmol/l was 2.6%.

## Discussion

This case suggests that closed‐loop insulin delivery can achieve safe glucose control in a hospitalized person with complex comorbidities during their terminal illness.

Closed‐loop systems deliver insulin in a glucose‐responsive manner, accommodating highly variable day‐to‐day insulin requirements, which are commonly seen during clinical deterioration and as people approach the end of life. Safe glucose control, avoiding hypoglycaemia and symptomatic hyperglycaemia can be difficult to achieve with standard subcutaneous insulin therapy in this clinical setting. Personalized glucose targets can be applied to the closed‐loop algorithm, allowing higher glucose levels while still maintaining adequate glucose control to prevent symptomatic hypo‐ or hyperglycaemia.

Closed‐loop technology may be a feasible option to reduce the burden of glucose management for people with diabetes who require insulin towards the end of life. Factory‐calibrated continuous glucose monitors require no calibrations, making this approach less invasive than standard finger‐stick capillary blood glucose monitoring; however accuracy of non‐calibrated continuous glucose monitors in the inpatient setting, and in those approaching the end of life, has not been evaluated. Insulin delivery via subcutaneous insulin pump is also less intrusive than multiple daily insulin injections.

Closed‐loop insulin delivery offers a novel approach to glucose management for people with diabetes who require insulin towards the end of life, and may be safer and less burdensome than standard insulin therapy. Acceptability of this technology to people with diabetes, family members and healthcare professionals at this time needs to be determined, and trials of safety and efficacy in this population undertaken.

## Funding sources

The randomized controlled trial was funded by Diabetes UK (#14/0004878) and Swiss National Science Foundation (P1BEP3_165297). Additional support for the study was provided by the National Institute for Health Research Cambridge Biomedical Research Centre and a Wellcome Trust Strategic Award (100574/Z/12/Z). Abbott Diabetes Care supplied discounted continuous glucose monitoring devices, sensors and details of communication protocol to facilitate real‐time connectivity.

## Competing interests

S.H. serves as a consultant for Novo Nordisk and for the ONSET group, and reports having received speaker/training honoraria from Medtronic. M.E.W. reports patents and patent applications related to closed‐loop. M.L.E. reports having received speaker honoraria/ conference travel support from Abbott Diabetes Care, Novo Nordisk, Astra Zeneca, MSD and Eli Lilly, and serving on advisory panels for Novo Nordisk, Abbott Diabetes Care, Medtronic, Roche, Dexcom and Cellnovo. R.H. reports having received speaker honoraria from Eli Lilly and Novo Nordisk, serving on advisory panel for Eli Lilly and Novo Nordisk, receiving license fees from B. Braun and Medtronic, having served as a consultant to B. Braun, and patents and patent applications related to closed‐loop. C.B., L.B., A.P.C. and C.S. declare no duality of interest associated with this manuscript.
